# Preliminary Comparison of a Modified cfDNA Extraction Protocol for Y-Chromosome Marker Detection in Maternal Plasma

**DOI:** 10.3390/diagnostics16121849

**Published:** 2026-06-15

**Authors:** Tugba Elgun, Yasemin Musteri Oltulu, Burcin Erkal Cam, Halil Ibrahim Arslan, Fulya Ozkal Molla, Pınar Ata, Asiye Gok Yurttas

**Affiliations:** 1 Department of Medical Biology, Faculty of Medicine, Biruni University, Istanbul 34015, Türkiye; telgun@biruni.edu.tr (T.E.); yoltulu@biruni.edu.tr (Y.M.O.); 2 Biruni University Research Center (B@MER), Biruni University, Istanbul 34015, Türkiye; halila@biruni.edu.tr; 3 Department of Molecular Biology and Genetics, Faculty of Arts and Sciences, Yıldız Technical University, Istanbul 34349, Türkiye; burcinerkall@gmail.com; 4 Department of Obstetrics and Gynecology, Faculty of Medicine, Biruni University, Istanbul 34015, Türkiye; fmolla@biruni.edu.tr; 5 Department of Medical Genetics, Faculty of Medicine, Marmara University, Istanbul 34722, Türkiye; pinar.ata@marmara.edu.tr; 6 Department of Biochemistry, Faculty of Medicine, Istanbul Atlas University, Istanbul 34403, Türkiye

**Keywords:** noninvasive prenatal diagnosis, TPY method, total cfDNA extraction from maternal plasma, real-time PCR, SRY, DYS14

## Abstract

**Objectives**: Noninvasive prenatal testing relies on the analysis of total cell-free DNA (cfDNA) in maternal plasma, where fetal-derived DNA constitutes only a minor fraction. This study aimed to preliminarily compare a modified TPY cfDNA extraction protocol with two commercial extraction kits for the downstream detection of Y-chromosome-specific markers in pregnancies carrying male fetuses. **Methods**: Plasma samples were obtained from 52 singleton pregnancies between 10 and 30 weeks of gestation with male fetal sex confirmed by ultrasonography. Total cfDNA was extracted from aliquots of the same maternal plasma samples using the modified TPY protocol, the QIAamp DSP Virus Kit, and the MagMAX™ Cell-Free DNA Isolation Kit. Quantitative real-time PCR was performed for the Y-chromosome-specific markers SRY and DYS14. At the same time, GLO was used as a reference marker to reflect the total cfDNA background. Extraction performance was assessed primarily using total cfDNA concentration and Ct values obtained from amplification of fetal-specific Y-chromosome markers. **Results**: Total cfDNA concentrations varied among the extraction methods, with the commercial kits yielding higher total cfDNA concentrations than the modified TPY protocol. In contrast, the TPY protocol yielded slightly lower mean Ct values for SRY and DYS14 than the commercial kits. SRY and DYS14 amplification was detected in 90.4% and 94.2% of samples, respectively. However, these Ct differences should be interpreted cautiously because fetal fraction, maternal DNA contamination, extraction recovery, and fragment size distribution were not directly measured. **Conclusions**: The modified TPY protocol showed preliminary technical feasibility for extracting total cfDNA from maternal plasma and enabling downstream amplification of Y-chromosome-specific markers in male pregnancies. Nevertheless, the observed lower Ct values do not establish selective fetal DNA enrichment, reduced maternal DNA contamination, or clinical superiority over commercial methods. Further analytical validation using standardized fetal fraction measurement, recovery efficiency testing, fragment size analysis, fetal-to-maternal DNA ratio assessment, and larger cohorts including both male and female pregnancies is required before broader clinical applicability can be determined.

## 1. Introduction

Recent technological advancements have enabled the early identification of diseases and improved the reliability of diagnostic processes. These developments have also led to significant progress in prenatal diagnostic approaches. In this context, establishing an early, rapid, and reliable diagnosis is essential for guiding appropriate clinical management decisions [1]. One of the major challenges in prenatal diagnostics is the inability of clinicians to access or interact with the fetus directly. Therefore, specialized imaging and analytical techniques are required for diagnosis, monitoring, and clinical decision-making. Traditionally, invasive procedures such as amniocentesis and chorionic villus sampling have been widely used; however, these methods carry potential risks for both the mother and the fetus. Consequently, there has been a growing interest in non-invasive diagnostic approaches among clinicians and patients in recent years [2]. Among these approaches, the analysis of cell-free fetal DNA (total cfDNA) in maternal plasma has emerged as one of the most widely used and clinically relevant methods. From approximately the 10th week of gestation, total cfDNA fragments circulating in maternal blood can be analyzed, providing early genetic information about the fetus. The primary sources of total cfDNA are believed to include the placenta, fetal tissues, and fetal hematopoietic cells. The release of total cfDNA into maternal circulation is thought to occur through mechanisms such as apoptosis, necrosis of placental and fetal cells, and active secretion processes [3,4]. Advances in the isolation of total cfDNA from maternal plasma and the development of non-invasive prenatal testing (NIPT) strategies represent major milestones in modern prenatal diagnostics. Within this framework, the ability to obtain and analyze fetal DNA from maternal blood without invasive procedures is critical for both clinical practice and research [4,5].

It is well established that non-invasive prenatal testing relies on the efficient isolation of total cell-free DNA (cfDNA), within which the fetal fraction typically constitutes a small proportion (approximately 1–4%). Accurate discrimination of this fraction is essential for reliable analysis. These approaches are currently used for chromosomal abnormalities such as trisomy 21, trisomy 18, trisomy 13, sex chromosome aneuploidies, and other pregnancy-related conditions [6,7,8,9,10]. In this context, Y-chromosome-specific markers such as **SRY** and **DYS14** are widely used for confirming the presence of fetal DNA in maternal plasma, particularly in methodological validation and fetal sex determination studies. The detection of these sequences provides a highly specific indication of fetal origin, independent of maternal genomic background [9].

Based on these considerations, the accurate isolation and analysis of total cfDNA from maternal plasma remain critical challenges in the field. Therefore, the primary aim of this study was not merely to obtain sufficient quantities of fetal DNA but to evaluate the efficiency and analytical performance of a modified TPY isolation method within a prenatal diagnostic framework. In this study, performance was assessed not solely on absolute total cfDNA concentration but also on amplification efficiency (Ct values) and reproducibility relative to established commercial kits. We propose that the modified TPY method may offer potential advantages, including reduced processing time. However, its impact on maternal DNA contamination remains to be determined. These features may enhance both research applications and the future development of non-invasive prenatal diagnostic methodologies.

## 2. Methods

### 2.1. Sample Collection

According to the power analysis results, to statistically evaluate within-group differences at the *p* < 0.05 level with more than 80% power, the sample size was determined to be 30. Approval for the study was received from the Biruni University Clinical Research Ethics Committee (December 2021; 2015-KAEK-57-21-07). Blood samples (plasma) were obtained from 52 pregnant women who applied to the Obstetrics and Gynecology outpatient clinic of Biruni University Faculty of Medicine Hospital and met the inclusion criteria, and they were categorized according to trimester. Inclusion criteria for the study were: pregnancy between 10 and 30 weeks of gestation, singleton pregnancy with a fetus identified as male by ultrasonography (USG), and absence of maternal malignancy or autoimmune disease. Plasma samples from 5 males (positive control) and 5 females (negative control) were used for control purposes. Blood samples from the first-trimester pregnant women were taken at 10–13; those from the second-trimester pregnant women were taken at 14–25; and those from the third-trimester pregnant women were taken at 26–30 weeks—the maternal plasma was collected in 1.5 mL DNA-LoBind tubes (Eppendorf, Hamburg, Germany).

### 2.2. cfDNA Isolation

cfDNA was isolated from maternal plasma samples obtained across all three trimesters of pregnancy using the QIAamp DSP Virus Kit (Qiagen, Hilden, Germany), the MagMAX™ Cell-Free DNA Isolation Kit (A29319, Thermo Fisher Scientific, Wilmington, DE, USA), and a manually developed isolation method (TPY; acronym derived from the initials of the researchers who developed the protocol). To obtain high-purity DNA, RNA, and protein, the samples were subjected to a series of purification steps. To minimize maternal genomic DNA contamination, the lysis step was intentionally omitted. It was hypothesized that most maternal genomic DNA is contained within intact maternal cells and nuclei. Therefore, a low-speed centrifugation step (1000× *g* for 10 min at 4 °C) was performed to pellet intact cellular components, retaining cell-free DNA in the supernatant. Although low-speed centrifugation is generally considered insufficient to cause significant cellular fragmentation, minimal cell disruption cannot be entirely ruled out. Following centrifugation, enzymatic reactions were performed in the supernatant fraction. For each participant, maternal plasma was divided into three equal aliquots and transferred into separate 1.5 mL DNA LoBind tubes (Eppendorf), with each aliquot assigned to one of the three extraction methods (TPY, MagMAX™, and QIAamp DSP Virus Kit). Proteinase K (50 µL) and RNase (5 µL) were added and incubated at 56 °C for 5 min. Subsequently, 2 mL of ethanol (96–100%) was added for precipitation.

**Figure 1:**
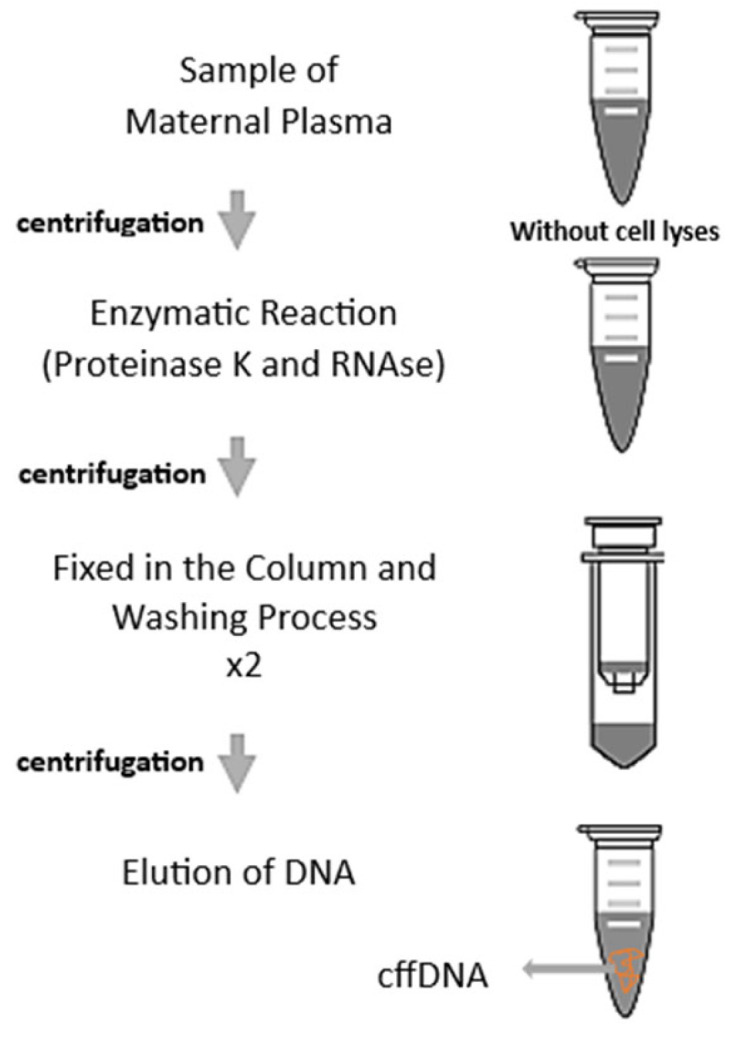
Total cfDNA extraction from maternal plasma for TPY.

cfDNA (including both maternal and fetal fractions) was bound to a silica membrane using a DNA-binding buffer. The washing step was performed three times using 500 µL wash buffer to reduce potential contaminants and PCR inhibitors that could affect downstream amplification analyses. Given that cfDNA fragments are shorter than genomic DNA, the washing procedure was carried out carefully to minimize potential loss. Finally, cfDNA was eluted from the silica membrane using 30 µL of elution buffer (Figure 1).

A quality-control criterion was defined based on the fetal fraction, a key determinant of analytical reliability in non-invasive prenatal testing. In the literature, the minimum fetal fraction required for reliable detection is generally reported to range between 2% and 4% [10]. Because fetal fraction was not quantified using a validated method, fetal fraction was not used as an inclusion criterion or analytical endpoint in the revised manuscript. Therefore, no fetal fraction threshold was applied in the final analysis. The extracted material was considered total cfDNA obtained from maternal plasma, which may contain both maternal and fetal fractions. The presence of fetal-derived DNA was assessed only through the amplification of Y-chromosome-specific SRY and DYS14 markers in pregnancies carrying male fetuses. Accordingly, the reported DNA concentrations represent total cfDNA concentrations rather than isolated total cfDNA concentrations.

**Figure 2:**
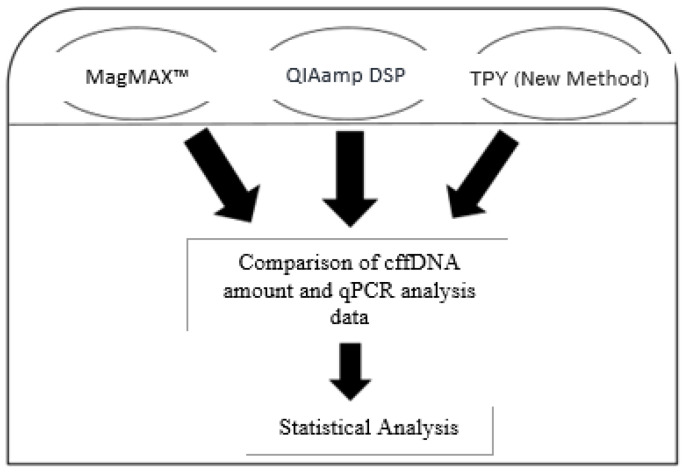
Analysis Diagram for the QIAamp DSP Virus Kit, MagMAX™ Cell-Free DNA Isolation Kit, and TPY.

The quantity and quality of the extracted DNA were assessed using a NanoDrop spectrophotometer (Thermo Fisher Scientific, Wilmington, DE, USA) and an Invitrogen™ Qubit™ 3 Fluorometer with the Qubit™ dsDNA HS Assay Kit (Thermo Fisher Scientific, Waltham, MA, USA). A 260/280 ratio of 1.8–2.0 (OD) indicates that the DNA was isolated in pure form. Attention was paid to ensure that the cfDNA amounts were above 8%. Plasma samples belonging to 5 men (positive control) and 5 non-pregnant women (negative control) were provided. Positive and negative control DNAs were isolated. DNA quantity was measured with Nanodrop (Figure 2).

### 2.3. Analysis with Real-Time Polymerase Chain Reaction

The BioRad CFX96 Touch Real-Time PCR (qPCR) Detection System was used for the qPCR analysis. First, 15 µL of the TaqMan-based PCR mix (P4600-100RXN, Sigma-Aldrich, Schnelldorf, Germany) was added to each well of the BioRad CFX96 multiwell plate, and 5 ng of DNA was added to it. The final volume in the wells was 20 µL. Initial denaturation of DNA at 95 °C for 5 min was followed by 36 cycles of denaturation at 95 °C for 10 s, annealing at 60 °C for 30 s, and extension at 72 °C for 10 s [10,11,12,13]. The minimum primer and probe concentrations were determined based on the cycle at which the reaction enters the exponential phase, as indicated by the Cycle Threshold (Ct). The maximum number of cycles for accepted Ct values is 36. Any reaction yielding Ct values > 36 was repeated; in our dataset, only a few reactions initially produced Ct values > 36, and the repeat analyses confirmed consistent Ct values below the threshold, underscoring the assay’s repeatability. The resulting Ct curves were evaluated separately for each sample in the FAM channel. Each sample was performed in triplicate for the three regions: SRY, DYS14, and GLO. GLO was selected as a reference marker because it is present in both maternal and fetal genomic DNA and therefore reflects the total cfDNA background in maternal plasma. In contrast, SRY and DYS14 were used as fetal-specific Y-chromosome markers in male pregnancies. The primers are as follows: forward: 5′ GTG CAC CTG ACT CCT GAG GAG 3′ and reverse: 5′ CCT TGA TAC CAA CCT GCC CAG 3′ with TAMRA-FAM probe of 5′ AAG GTG AAC GTG GAT GAA GTT GGT GG 3′. DYS14 regions are as follows: forward: 5′ CATCCAGAGCGTCCC TGG 30, reverse: 50 TTCCCCTTTGTTCCCCAAA 30 with TAMRA-FAM probe of 5′ CGAA GCCGAGCTGCCCATCA 3′. SRY regions are as follows: forward: 5′ TGG CGA TTA AGT CAA ATT CGC 3′, reverse: 5′ CCC CCT AGT ACC CTG ACA ATG TAT T 3′ with TAMRA-FAM probe of 5′ AGC AGT AGA GCA GTC AGG GAG GCA GA 3′.

### 2.4. Statistical Analysis

Differences in amplification levels were evaluated based on cycle threshold (Ct) values. Statistical analyses were performed using GraphPad Prism version 9.1.1 (GraphPad Software Inc., San Diego, CA, USA). For each sample, qPCR analyses were conducted in triplicate, and the mean Ct value of the technical replicates was calculated and used for statistical analysis. Technical replicates were used only to assess assay consistency and were not treated as independent biological observations.

Normality of Ct values and total cfDNA concentration data was assessed using the Shapiro–Wilk test. Because the same plasma samples were aliquoted and extracted using all three methods, comparisons among the extraction methods were performed using repeated-measures ANOVA followed by Tukey’s multiple comparisons test for normally distributed data. When the normality assumption was not met, the Friedman test followed by Dunn’s post hoc test was used. The level of statistical significance was set at *p* < 0.05.

Detection rates of SRY and DYS14 amplification were compared using Fisher’s exact test. Because only pregnancies carrying male fetuses were included, specificity, false-positive rate, receiver operating characteristic analysis, and diagnostic accuracy could not be evaluated. Therefore, the statistical analysis was limited to comparisons of total cfDNA concentration, Ct values, and marker detection rates among the extraction methods.

## 3. Results

The ages of the pregnant women ranged from 20 to 42 years, with a mean age of 33.1 years. Among the 52 samples, 18 were obtained from first-trimester pregnancies (gestational weeks 10–13), 20 from second-trimester pregnancies (gestational weeks 14–25), and 14 from third-trimester pregnancies (gestational weeks 26–30). Maternal body weight ranged from 54 to 89 kg, with a mean of 67.42 kg. The mean gestational age was 19.3 ± 1.6 weeks.

It was the first pregnancy for 57.7% (30/52) of the pregnant women in this study. There was a history of abortion (miscarriage) in 15.4% (8/52) of the pregnant women. With the developed protocol, total cfDNA was 3.542 ± 0.89 ng/μL in the first trimester, 4.191 ± 1.127 ng/μL in the second trimester, and 6.273 ± 1.641 ng/μL in the last trimester. The amounts of total cfDNA with MAG-MAX were determined as 4.436 ± 1.11 ng/μL in the first trimester, 5.639 ± 1.14 ng/μL in the second trimester, and 7.81 ± 1.57 ng/μL in the last trimester. The amounts of total cfDNA with DSP-VK were determined as 5.193 ± 0.72 ng/μL in the first trimester, 6.955 ± 0.86 ng/μL in the second trimester, and 8.47 ± 0.691 ng/μL in the last trimester (Table 1). Quantitative analyses revealed that the total cfDNA yields obtained using the MagMAX™ and QIAamp DSP Virus Kits were not statistically different (ANOVA, *p* > 0.05). Although the modified TPY method yielded slightly lower cfDNA concentrations, its qPCR amplification efficiency (i.e., lower mean Ct values for fetal-specific markers) supports its potential utility.

In the literature, the fetal fraction typically accounts for approximately 3–6% of total circulating cfDNA [13]. Variations in absolute concentration values (e.g., 0.05–0.6 ng/μL reported in some studies) may be attributed to differences in extraction protocols and quantification methods [13,14,15].

Quantitative analysis showed that total cfDNA concentrations differed among the extraction methods. The commercial kits yielded higher total cfDNA concentrations than the modified TPY protocol, whereas the MagMAX™ and QIAamp DSP Virus Kits showed statistically comparable yields. Following qPCR analysis, successful amplification was defined as a Ct value below 36. Based on this criterion, SRY was successfully amplified in 47 of 52 plasma samples (90.4%), whereas DYS14 was detected in 49 of 52 samples (94.2%). These percentages represent binary detection outcomes, namely the presence or absence of amplification, and no statistically significant difference was observed between the detection rates of SRY and DYS14 (Fisher’s exact test, *p* = 0.8885).

In contrast to these binary detection outcomes, the study’s main comparative observation was based on continuous Ct values. The modified TPY protocol yielded lower mean Ct values for both SRY and DYS14 compared with the commercial kits (*p* < 0.05). However, higher total cfDNA yield obtained with commercial kits and lower Ct values obtained with TPY are not necessarily contradictory because total cfDNA concentration and fetal-specific Y-chromosome marker amplification represent different analytical outputs. Therefore, the lower Ct values observed with TPY should be interpreted as differences in downstream amplification signal rather than direct evidence of increased fetal fraction, improved fetal DNA recovery, reduced maternal DNA contamination, or selective fetal DNA enrichment. Additional validation parameters would be required to support such conclusions.

According to the amplification analysis, the mean Ct values for the SRY gene were 32.03 with the MagMAX kit and 30.34 with the QIAamp DSP Virus Kit, while the corresponding values for DYS14 were 30.19 and 29.95, respectively. In comparison, the modified TPY protocol yielded mean Ct values of 29.83 for SRY and 29.84 for DYS14 (Table 2).

## 4. Discussion

The discovery of total cfDNA fragments in maternal plasma has significantly advanced the field of noninvasive prenatal diagnostics. In particular, early fetal sex determination has become clinically important for the management of X-linked genetic disorders such as hemophilia and Duchenne muscular dystrophy, where early diagnosis can guide clinical decision-making [15,16]. Compared with invasive procedures such as chorionic villus sampling and amniocentesis, noninvasive approaches require highly sensitive and reliable analytical methods to ensure clinical applicability. In recent years, aspects such as cost-effectiveness, clinical utility, and target populations for total cfDNA-based testing have been widely discussed in the literature [16,17]. It is well established that amplification methodology is a critical determinant of assay performance. Studies have demonstrated that probe-based qPCR assays provide higher specificity compared to conventional primer-based approaches [17,18]. In this context, the present study aimed to develop an isolation protocol that yields total cfDNA of sufficient quality and quantity for reliable downstream analysis.

In the present study, maternal plasma samples from 52 pregnancies in which fetuses were identified as male by ultrasonography were analyzed across gestational stages. Total cfDNA was successfully isolated using the modified TPY protocol. Commercial kits, including the QIAamp DSP Virus Kit and the MagMAX™ Cell-Free DNA Isolation Kit, were used as reference methods for comparison. The detection of the Y chromosome confirmed the presence of fetal DNA-specific markers, namely SRY and DYS14. These markers are widely used in the literature for both methodological validation and fetal sex determination due to their high specificity for fetal-derived DNA.

Amplification of SRY and DYS14 was achieved in 90% and 94% of samples, respectively, and postnatal data confirmed male sex in 96% of cases. In addition, complete concordance was observed between the commercial kits and postnatal outcomes. While the modified TPY protocol yielded slightly lower Ct values than the commercial kits, this finding should be interpreted with caution. Lower Ct values may reflect improved amplification efficiency; however, they do not, by themselves, establish superior diagnostic performance without comprehensive validation of fetal fraction and contamination levels. The consistently lower Ct values observed for DYS14 compared to SRY are likely attributable to the multicopy nature of the DYS14 region on the Y chromosome. This characteristic enhances detection sensitivity but does not necessarily confer higher diagnostic accuracy, underscoring the importance of interpreting these markers in their genomic context. It is important to emphasize that lower Ct values should not be interpreted as direct evidence of selective fetal DNA enrichment or reduced maternal DNA contamination. In the present study, the commercial kits yielded higher total cfDNA concentrations, whereas the modified TPY protocol produced slightly lower Ct values for SRY and DYS14. These findings are not necessarily contradictory, because total cfDNA concentration and fetal-specific Y-chromosome marker amplification represent different analytical outputs. Several technical factors, including differences in inhibitor removal, elution composition, DNA fragment accessibility, extraction bias, or compatibility of the extracted material with downstream qPCR amplification may influence lower Ct values. Therefore, the observed Ct differences should be interpreted as preliminary evidence of differential downstream amplification performance rather than proof of improved fetal DNA recovery. Previous studies support the importance of fragment size and isolation efficiency in total cfDNA analysis. For example, Jain et al. (2019) demonstrated that shorter DNA fragments are enriched for fetal DNA, thereby reducing test failure rates [6,18,19]. Similarly, Pedini et al. (2021) reported that differences in isolation techniques can significantly affect DNA yield, fragment size distribution, and detectability [5]. These findings are consistent with our observations and highlight the critical role of isolation methodology in total cfDNA-based applications.

In addition, previous studies have emphasized the importance of isolating methylated total cfDNA to improve fetal DNA detection and distinguish it from maternal DNA [20,21]. While our study did not focus on methylation-based discrimination, the high amplification success observed supports the proposed method’s potential applicability in clinical and research settings [20,21]. Earlier work also demonstrated that isolation techniques significantly influence DNA quality, with magnetic bead-based methods showing advantages in purity and sensitivity [22]. Together, these studies underline the importance of optimizing isolation strategies to obtain high-quality fetal DNA [21,22].

Despite these findings, several limitations of the present study should be acknowledged. First, the inclusion of only male fetuses limits the ability to assess diagnostic specificity and generalizability to broader clinical populations. Second, using only two Y-chromosome markers restricts the scope of validation. Third, fetal fraction estimation was performed using a semi-quantitative approach, which requires further standardization. Finally, the study was designed as a methodological comparison rather than a full diagnostic validation study; therefore, parameters such as sensitivity, specificity, and predictive values were not comprehensively evaluated. Overall, the findings of this study suggest that the modified TPY isolation method provides a reliable and reproducible approach for total cfDNA extraction, with performance comparable to that of established commercial kits. Future studies involving larger, more diverse patient populations, additional genetic markers, and standardized fetal fraction assessment methods are required to further validate and expand the clinical applicability of this approach.

***Limitation:*** The study was designed as a methodological comparison rather than a full analytical validation study; therefore, parameters such as fragment-size distribution, analytical precision, extraction efficiency, and quantification of the comprehensive fetal fraction were not systematically evaluated. In addition, maternal DNA contamination, fetal fraction, fetal-to-maternal DNA ratio, extraction recovery efficiency, fragment size distribution, limit of detection, linearity, and intra- or inter-run analytical precision were not systematically evaluated. Therefore, the present study should be considered a preliminary technical comparison rather than a full analytical or clinical validation study. These limitations prevent any definitive conclusion regarding superiority, equivalence, selective fetal DNA enrichment, or reduced maternal DNA contamination of the modified TPY protocol compared with commercial kits.

## 5. Conclusions

In conclusion, the modified TPY method demonstrated a reliable and reproducible approach for the isolation of total cfDNA from maternal plasma, showing performance comparable to that of established commercial kits. The method yielded consistent amplification results and may offer practical advantages such as reduced processing time. However, potential effects on maternal DNA contamination were not evaluated in the present study and require further investigation.

However, the findings of this study should be interpreted in light of its limitations. These findings should be interpreted within the methodological scope of the study. The inclusion of only male fetuses and the use of a limited number of Y-chromosome markers restrict the generalizability of the results. In addition, fetal fraction estimation was performed using a semi-quantitative approach, and comprehensive diagnostic performance metrics were not evaluated.

Therefore, while the modified TPY protocol appears to be a promising alternative for total cfDNA extraction from maternal plasma, further validation in larger and more diverse cohorts, including both male and female pregnancies and additional genetic markers, is required. Future studies incorporating standardized fetal fraction assessment and full diagnostic accuracy analyses will be essential to determine its broader clinical applicability. The present findings should be interpreted as a methodological proof of concept rather than a fully validated diagnostic approach.

## Figures and Tables

**Table 1:** Comparison of total cfDNA concentrations (ng/µL, mean ± SD) obtained using three different extraction methods across trimesters.

Pregnant in the Trimester (Weeks)	cff-DNA (ng/μL)		
	TPY (New Method)	MAG-MAX	QIAamp DSP Virus Kits
**10–12**	3.542 ± 0.89	4.436 ± 1.11	5.193 ± 0.72
**13–26**	4.191 ± 1.127	5.639 ± 1.14	6.955 ± 0.86
**>27**	6.273± 1.641 *	6.197 ± 1.557	8.47 ± 0.691

* *p* < 0.05; one-way ANOVA.

**Table 2:** Amplification data obtained with the kits and the developed method.

Analysis	SRY Average Ct Value	DYS14 Average Ct Value
**GLO**	25.32	25.47
**Positive Control**	26.082	25.223
**Negative Control**	-	-
**Mag-Max**	32.03	30.19
**DSP-VK**	30.34	29.95
**TPY (new method)**	29.83 *	29.835 *

* *p* < 0.05; Tukey’s multiple comparisons test.

## Data Availability

The original contributions presented in this study are included in the article. Further inquiries can be directed to the corresponding author.
